# Research progress of tertiary lymphoid structure in hepatocellular carcinoma: from prognostic biomarkers to new strategies for immunotherapy

**DOI:** 10.3389/fimmu.2026.1741542

**Published:** 2026-04-27

**Authors:** Shimin Yuan, Xianting Luo, Shu Li, Long Cao, Xiang Chen, Ling Wang, Yan Liu, Zipeng Li, Tongnuo Wu, Hongcai Fang, Mingfu Tong

**Affiliations:** 1 Department of Hepatobiliary Surgery, Jiujiang University Affiliated Hospital, Jiujiang, China; 2 Jiangxi Medical College of Nanchang University, Nanchang, Jiangxi, China

**Keywords:** hepatocellular carcinoma, immunotherapy biomarker, non-invasive testing, tertiary lymphoid structure, tumor immune microenvironment

## Abstract

Tertiary lymphoid structures (TLS) are important ectopic immune aggregates that develop within the tumor microenvironment (TME) of hepatocellular carcinoma (HCC) and have attracted increasing attention due to their potential clinical value. Recent studies have identified TLS as key players in prognosis assessment and immunotherapy response prediction in HCC, with their maturity and spatial distribution being critical determinants of clinical outcomes. TLS can be classified into intratumoral and peritumoral types, which exhibit distinct predictive values; the clinical significance of peritumoral mature TLS remains controversial. Histopathological analysis remains the gold standard for detecting TLS structure and composition. In recent years, predictive models based on CT/MRI radiomics and machine learning have achieved promising progress in non-invasive detection. This review summarizes and analyzes the formation mechanism of TLS in HCC, emphasizes their structural characteristics, spatial distribution, clinical value, and detection methods, presents current challenges and research directions, and provides a new perspective for the diagnosis and treatment of HCC.

## 1 Introduction

Primary liver cancer is a common malignancy worldwide, with hepatocellular carcinoma (HCC) accounts for the majority of cases (1). HCC is the sixth most common cancer globally and the second leading cause of cancer-related deaths in China (2). Due to its insidious onset, approximately 70% of patients are diagnosed at intermediate or advanced stages, losing the opportunity for curative resection, and the overall 5-year survival rate remains only about 18% (3). Surgical recurrence rates within five years are as high as 70%, and donor shortages severely limit the widespread application of liver transplantation (4–6).

The tumor microenvironment (TME) plays a critical role in HCC progression and treatment response. For advanced HCC, systemic therapy has evolved from the era of single-agent tyrosine kinase inhibitors to a new phase of “targeted therapy combined with immunotherapy”, with multiple combination regimens now listed as first-line options in international guidelines (7, 8). Despite these advances, the objective response rate (ORR) remains only 20% - 30%, and median progression-free survival (mPFS) is generally less than 7 months, indicating that a substantial proportion of patients do not benefit from current treatments (9, 10). The heterogeneity of immune checkpoint inhibitor (ICI) efficacy is a core challenge in clinical practice. The unique immunosuppressive microenvironment of HCC—characterized by PD-L1-high tumor-associated macrophages (TAMs), enriched regulatory T cells (Tregs), and myeloid-derived suppressor cells (MDSCs)—forms physical and functional barriers that restrict effector T cell infiltration and activation (11, 12). Additionally, low tumor mutational burden (TMB) and weak antigen presentation capacity result in an ORR of only approximately 15% for ICI monotherapy (13, 14). In this context, the identification of biomarkers that can accurately predict prognosis and immunotherapy response has become critical for advancing individualized treatment strategies in HCC.

**Figure 1:**
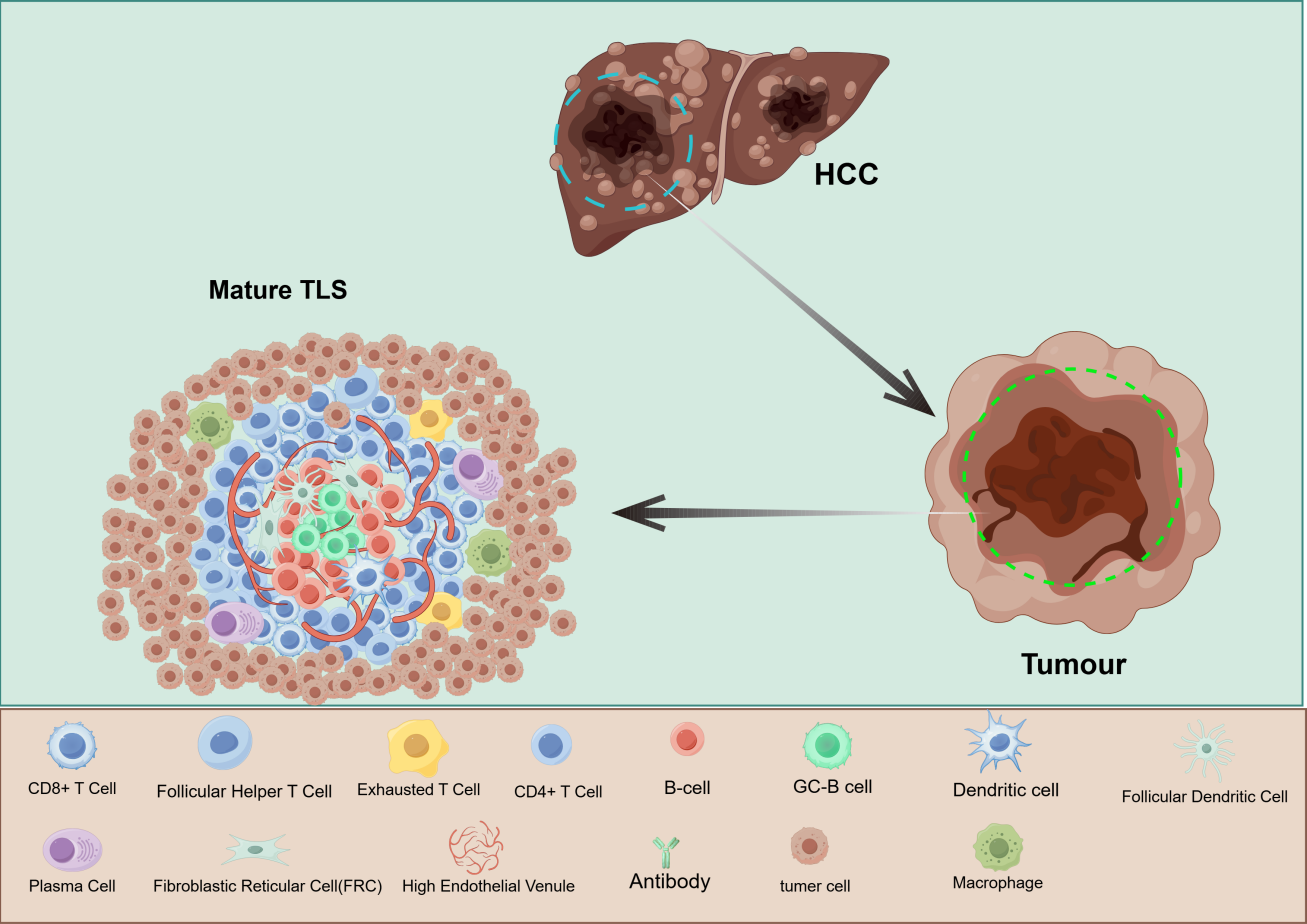
Model diagram of tertiary lymphoid structure in the microenvironment of hepatocellular carcinoma. This figure shows the core composition and spatial layout of a mature TLS within an HCC tumor: the center is a B-cell follicle (orange), containing a germical center (green); Beside the follicles is the T-cell area (light blue), rich in CD4^+^ and CD8^+^ T cells, and scattered regulatory T cells can be seen (red). Dendritic cells (blue and white) are distributed at the junction of the two zones and around the T-cell zone, responsible for capturing and cross-presenting tumor antigens. High endothelial venules (HEV, red) present as high cubic endothelium, express PNAd, serve as the main gateway for peripheral immune cells to enter TLS, and form a radially arranged vascular network along the edge of the T cell region. The figure was created by Figdraw. (http://www.figdraw.com).

In recent years, tumor-associated tertiary lymphoid structures(TLS), specialized immune aggregates formed within the TME, have demonstrated significant prognostic and predictive value across various solid tumors (13). TLS are ectopic lymphoid organs that develop in non-lymphoid tissues under pathological conditions such as chronic inflammation, autoimmune diseases, or cancer (14). Unlike conventional secondary lymphoid organs, TLS lack a capsule and afferent lymphatic vessels but contain well-developed T-cell zones, B-cell follicles, dendritic cells, and high endothelial venules (HEVs), thereby forming localized and efficient “immune response hubs” (15–18) (**Figure 1**). Mature TLS in HCC—characterized by enrichment of CD20^+^ B cells and CD8^+^ T cells—serve as positive prognostic markers and are significantly associated with higher ORR to ICIs and lower postoperative recurrence rates. Conversely, the absence or structural immaturity of TLS, particularly the lack of germinal center-like structures, correlates with primary resistance to immunotherapy (19–21). These findings suggest that intratumoral mature TLS not only represent favorable prognostic indicators but also closely correlate with responses to ICIs and combination regimens.

Despite these advances, several key unresolved questions remain: (1) the mechanistic distinction between mature and immature TLS and their respective functional roles in local antigen presentation and B-cell activation; (2) the controversial prognostic value of peritumoral TLS, with conflicting results across studies; (3) the lack of standardized detection methods and consensus definitions for TLS classification; and (4) the potential of TLS as a therapeutic target that can be modulated to enhance antitumor immunity. Non-invasive prediction of intratumoral TLS using MRI-based radiomics has emerged as a promising approach, with recent studies demonstrating feasibility (22, 23). Mechanistically, mature TLS have been shown to enrich TCF1^+^PD1^+^CD8^+^ T cells, a stem cell-like exhausted T cell population that is essential for sustaining antitumor immune activation (24). This review aims to: (i) comprehensively synthesize the formation mechanisms of TLS in HCC; (ii) critically analyze the clinical significance of TLS spatial distribution and maturity; (iii) evaluate emerging non-invasive detection methods; and (iv) propose a roadmap for future research priorities to facilitate the translation of TLS from a prognostic biomarker to a clinically actionable tool for immunotherapy stratification.

## 2 Formation mechanisms of TLS

The formation of TLS is an active process driven by chronic inflammation or sustained antigen exposure, a phenomenon termed “lymphoid neogenesis” (25, 26). This process initiates when tissue-resident innate immune cells, such as inflammatory monocytes/macrophages and dendritic cells, recognize danger signals including viral antigens and tumor-associated antigens, leading to their activation and secretion of lymphotoxin (LTα/β) (27, 28). Membrane-bound LTα1β2 binds to the lymphotoxin β receptor (LTβR) on the surface of stromal cells, including fibroblasts and endothelial cells. This interaction activates both classical and non-classical NF-κB signaling pathways (29). Consequently, stromal cells upregulate adhesion molecules (e.g., VCAM-1, ICAM-1) and key chemokines such as CXCL13, CCL19, and CCL21. These molecules establish a “homing code” that facilitates the recruitment, homing, and retention of lymphocytes, thereby initiating TLS formation (30, 31).

**Figure 2:**
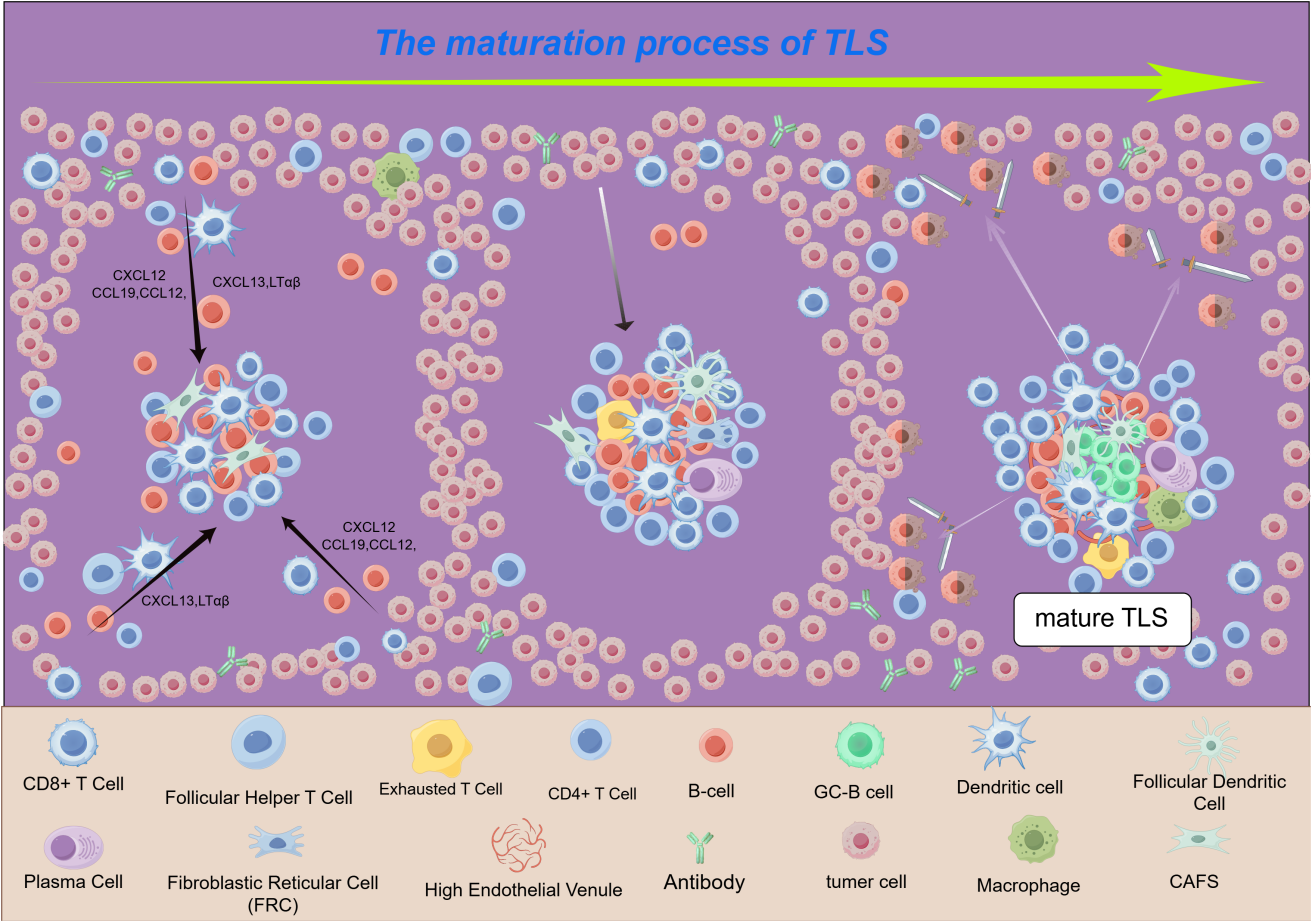
HCC-TLS “maturity timeline” model diagram (left → right). ① Initiation: CXCL13^+^CAF/DC recruits B and T cells; ② Assembly: Fibroblasts secrete extracellular matrix (ECM) to support the T-cell region (blue) and B-cell region (orange-red), and lymphocytes diffuse and aggregate. ③ Maturation: A TLS with a germinal center (GC, light green) is formed to attack the surrounding tumors, while Treg/Breg (gray shield) forms a balance. The entire maturation process takes approximately 3 weeks and overlaps with the anti-PD-1 therapeutic window. The figure was created by Figdraw. (http://www.figdraw.com).

Following the initial recruitment, lymphocyte aggregates undergo a series of ordered maturation stages. Under the guidance of the CXCL13-CXCR5 axis, T follicular helper (Tfh) cells and B cells are recruited to the same area, forming the rudimentary B-cell follicles (32, 33). Concurrently, the CCL19/CCL21-CCR7 axis recruits T cells and dendritic cells to establish the T-cell zone, together constituting a pre-TLS structure (34, 35). At this stage, interleukin-7 (IL-7) exerts multiple pro-formation effects by binding to its receptor (IL-7R) (36, 37): it supports lymphocyte survival and proliferation, upregulates LTα/β expression, and synergistically enhances CCL19/CCL21 production, thereby consolidating TLS architecture. Under sustained LTβR signaling, local endothelial cells differentiate into high endothelial venules (HEVs). HEVs specifically express peripheral node addressin (PNAd), which serves as the exclusive entry point for circulating lymphocytes to directly enter TLS, greatly enhancing the efficiency of local immune responses (38). Fully functional TLS contain germinal centers that support B-cell affinity maturation and plasma cell differentiation, while the T-cell zone facilitates antigen presentation and effector T-cell activation, rendering TLS an efficient *in situ* “immune battlefield” (**Figure 2**).

The maturation of TLS is regulated by a complex interplay of positive and negative factors. Positive regulators include Tfh cells, which provide critical help for B-cell activation and germinal center formation; B cells themselves, which contribute to the structural integrity of TLS; follicular dendritic cells (FDCs), which are essential for antigen presentation and germinal center maintenance; LTβR signaling; and the CXCL13-CXCR5 axis. Conversely, negative regulators have recently emerged as important modulators. Using spatial transcriptomics, Tang and colleagues recently reported that upregulation of the tryptophan-metabolizing enzyme TDO2 in liver tumor cells was associated with inhibition of TLS maturation, and blocking TDO2 appeared to reshape the immune microenvironment and, in combination with anti-PD-1 therapy, improve TLS maturity and antitumor immunity in preclinical models (39). These findings, while promising, are derived from a single study and require independent validation. Immunosuppressive signals, such as those mediated by regulatory T cells and certain metabolic pathways, may also impede TLS development.

In the specific context of HCC, persistent inflammatory stimuli, including HBV/HCV infection, alcoholic liver disease, and non-alcoholic steatohepatitis, promote the expression of lymphoid chemokines such as CXCL13, CCL19, and CCL21,which recruit B cells, T cells and dendritic cells into the TME (40, 41). Following local aggregation, these cells gradually induce TLS formation through intercellular interactions and LTβR pathway activation (42, 43). Notably, therapeutic interventions can actively induce TLS. Hepatic artery infusion chemotherapy (HAIC) combined with the FOLFOX regimen significantly enhances TLS formation and maturation in HCC (44). This induction may be attributed to immunogenic cell death (ICD) triggered by chemotherapeutic agents, which releases tumor antigens and activates local immune responses. Similarly, neoadjuvant ICI therapy has been associated with high-density intratumoral TLS in HCC, suggesting that TLS may serve as both a predictive marker and a contributing factor for immunotherapy response (20).

Non-cellular components in the TME, including intratumoral bacteria and metabolic remodeling, have also been suggested to be involved in TLS formation, although evidence remains limited. In HCC, emerging data indicate that the presence of intratumoral bacteria may correlate with infiltration of TLS-associated immune cells (45), potentially activating immune cells via pattern recognition receptors (e.g., Toll-like receptors, TLRs) and thereby contributing to TLS development. Further studies are needed to confirm this association and to determine causality. Additionally, emerging evidence points to a potential role for metabolic pathways, such as glutathione synthesis, play a role in TLS formation. Arai and colleagues observed that glutathione accumulates significantly in TLS, and inhibition of the cystine/glutamate transporter prevents TLS formation in a kidney disease model (46), suggesting that metabolic reprogramming might represent one underlying mechanism. Whether similar mechanisms operate in HCC remains to be investigated. The possible involvement of metabolic regulation in TLS formation has been further supported by recent studies, but much of this evidence remains preliminary and requires validation in larger cohorts, preferably in HCC-specific contexts.

## 3 Clinicopathological significance of TLS in liver cancer

The clinicopathological significance of TLS in HCC has garnered increasing attention, with their relationship to patient prognosis, immunotherapy response, and immune microenvironment exhibiting a complex “double-edged sword” effect. This complexity primarily stems from the heterogeneity in the spatial distribution (intratumoral versus peritumoral) and maturity (presence or absence of germinal centers). A summary of TLS classification by location, maturity, defining features, and clinical relevance is provided in **Table 1**.

**Table 1:** Classification of tertiary lymphoid structures in hepatocellular carcinoma.

Type	Location	Maturity	Defining features	Clinical relevance
Intratumoral TLS (iTLS)	Within tumor parenchyma	Mature	Germinal centers, BCL6^+^, CD21^+^ FDC networks, HEV integrity	Favorable prognosis; higher ICI response rate; lower recurrence risk
		Immature	Lymphocyte aggregates without germinal centers, absent or immature HEV	Limited or no prognostic value; may lack functional antitumor activity
Peritumoral TLS (pTLS)	Adjacent to tumor margin, within surrounding stroma	Mature	Germinal centers present, organized lymphoid architecture	Controversial; may correlate with either favorable or poor prognosis depending on immune composition
		Immature	Disorganized lymphocyte aggregates, no germinal centers	Often associated with immunosuppressive microenvironment; generally poor prognostic value

TLS, tertiary lymphoid structures; FDC, follicular dendritic cells; HEV, high endothelial venules; ICI, immune checkpoint inhibitor.

### 3.1 Prognostic significance of TLS spatial distribution

Multiple studies have consistently demonstrated that the presence of intratumoral TLS (iTLS) is significantly associated with improved survival in HCC patients. Using TCGA-LIHC cohort analysis, Jia et al. showed that high iTLS density correlated with better prognosis and significantly improved disease-free survival (DFS) (47). In a recent study, Ahn and colleagues analyzed clinicopathological data from 191 surgically resected HCC cases, including 160 conventional HCC and 31 lymphocyte-rich HCC (LR-HCC) cases. Multi-omics analysis revealed that LR-HCC exhibited better prognostic outcomes compared with conventional HCC, and TLS were the characteristic structures frequently observed in LR-HCC, suggesting that TLS represent an effective antitumor immune response (48). Furthermore, a multicenter study including Huashan Hospital affiliated with Fudan University demonstrated that tumors lacking TLS showed excessive mTOR signaling pathway activation and uncontrolled cell cycle progression. Notably, in HCC patients undergoing liver transplantation, the absence of intratumoral TLS abundance emerged as an independent risk factor for poor prognosis, associated with higher recurrence risk and shorter survival (49).

In contrast, the clinical significance of peritumoral TLS (pTLS) remains highly controversial. High pTLS density may be associated with a distinct immune pattern, but its prognostic value is unclear and may even correlate with poor prognosis in certain contexts. Some studies suggest that pTLS may enrich regulatory immune cells, thereby creating a relatively immunosuppressive microenvironment (50). Conversely, other studies, including work by Li and Long’s teams, have reported that high pTLS density may have positive prognostic and immunotherapy-predictive value in HCC (51, 52). This controversy underscores the complexity of the pTLS biology; pTLS may represent a heterogeneous entity rather than a uniform structure. Future research should focus on refined subtyping based on internal immune cell composition, maturity status, and spatial relationship with tumor boundaries. To better understand these discrepancies, several factors warrant consideration. Differences in TLS maturity likely contribute significantly to divergent prognostic associations; mature pTLS with functional germinal centers may support antitumor immunity, whereas immature pTLS may lack this capacity. Variations in immune cell composition—such as enrichment of effector versus regulatory populations—may also explain conflicting findings. Additionally, tumor stage and etiology (e.g., viral hepatitis versus non-alcoholic steatohepatitis) may influence the functional orientation of pTLS. These observations highlight the need for standardized classification criteria and multi-parametric assessment in future studies.

### 3.2 Prognostic significance of TLS maturity

Recent studies indicate that TLS maturity in HCC is a key determinant of prognosis and therapeutic response, with prognostic value extending beyond simple presence-or-absence assessment. Mature iTLS, defined by the presence of germinal centers, high density, or structural integrity, have been established as independent favorable prognostic factors associated with lower recurrence risk and longer survival (53–55). Regarding immunotherapy response, following neoadjuvant immunotherapy (e.g., PD-1 inhibitors combined with chemotherapy), increased density of mature intratumoral TLS significantly correlated with pathological response rate and improved survival (20, 56). Lanickova and colleagues demonstrated that mature TLS enhance therapeutic response by enriching ICI-responsive TCF1^+^PD1^+^CD8^+^ T cells, a stem cell-like exhausted T cell population critical for sustaining effective antitumor immunity (24). Furthermore, therapeutic interventions can promote TLS maturation; a study from Sun Yat-sen University Cancer Center showed that HAIC combined with FOLFOX significantly promoted TLS maturation, improved the immune microenvironment, and prolonged progression-free survival in HCC patients (44).

The functional distinction between mature and immature TLS is critical. Mature TLS, characterized by well-organized germinal centers, support local antigen presentation, B-cell affinity maturation, and plasma cell differentiation, thereby fostering robust adaptive immune responses. In contrast, immature TLS lack these structures and functional capabilities, potentially failing to initiate effective antitumor immunity (54, 55). The identification of mature TLS requires demonstration of germinal center formation, HEV integrity, and organized lymphoid aggregation. Immature TLS, lacking these components, exhibit limited prognostic value and may even be functionally insufficient.

Therefore, the evaluation of TLS should shift from a binary (presence/absence) to a spectral (immature → mature) continuous evaluation system. Combining multi-dimensional assessment of TLS location (intratumoral versus peritumoral) and mature status can substantially optimize risk stratification and therapeutic decision-making for HCC patients (53–55, 57).

### 3.3 Tertiary lymphoid structures and immunotherapy response

The predictive value of TLS for immunotherapy outcomes has emerged as a promising area of investigation. Patients with high-density intratumoral TLS, particularly those with mature TLS, demonstrate significantly improved responses to immune checkpoint inhibitors (20, 56). Mechanistically, mature TLS serve as local hubs that facilitate the recruitment and activation of TCF1^+^PD1^+^CD8^+^ T cells, a population essential for sustained antitumor immunity (24). Additionally, TLS may enhance antigen presentation and promote the development of tumor-specific T-cell responses within the TME.

In neoadjuvant settings, TLS formation and maturation have been observed following ICI treatment, suggesting a bidirectional relationship in which TLS both predict and contribute to therapeutic efficacy (20). Combination strategies that incorporate agents capable of inducing TLS, such as HAIC-FOLFOX, may further augment immunotherapy responses (44). However, it is important to note that current evidence is primarily derived from retrospective analyzes and small cohort studies; prospective validation in larger, well-controlled trials is urgently needed.

## 4 Detection and clinical application prospects of TLS

In HCC research and clinical practice, detection strategies for TLS encompass two main technical approaches: invasive and non-invasive.

### 4.1 Invasive detection

Invasive testing, based on histopathological analysis, relies on tumor specimens obtained via surgical resection or biopsy and is currently regarded as the gold standard for evaluating TLS structure and composition. Conventional hematoxylin and eosin (H&E) staining can identify lymphocyte aggregation areas, while multicolor immunohistochemistry or multiplex immunofluorescence techniques enable detailed analysis of TLS microarchitecture, including T-cell zones, B-cell follicles, germinal center formation, and the presence of high endothelial venules, thereby accurately determining maturity status (47, 53). However, these methods are limited by tumor spatial heterogeneity, which may introduce sampling bias. Additionally, invasive techniques cannot be implemented preoperatively and are ill-suited for continuous monitoring of TLS dynamic evolution, restricting their application in real-time clinical decision-making (22).

### 4.2 Non-invasive detection

In recent years, significant progress has been made in non-invasive detection technologies, enabling preoperative assessment of TLS. Radiomics and artificial intelligence models based on CT or MRI are current research hotspots. By extracting textural, morphological, and functional imaging features from tumors and surrounding areas and applying machine learning algorithms, researchers have achieved relatively accurate preoperative prediction of intratumoral TLS presence and maturity status. Long and colleagues developed a radiomics model based on a large multicenter cohort using machine learning methods to identify imaging features, including intratumoral heterogeneous enhancement and capsule integrity, that correlate with iTLS maturity; the model also demonstrated excellent discrimination for predicting pTLS density (51). Similarly, Li and colleagues developed a radiomics nomogram based on multi-parameter MRI features for non-invasive preoperative prediction of iTLS maturity in HCC, potentially informing individualized postoperative TKI-ICI treatment decisions (23).

Despite these advances, critical limitations must be acknowledged. Model reproducibility across different centers remains a concern, with most studies lacking external validation in independent cohorts. Dataset heterogeneity, including variations in imaging protocols, scanner types, and patient populations, may affect model generalizability. Furthermore, existing models exhibit weak detection capability for peritumoral TLS, which may reflect the greater biological complexity of this compartment. Brief comparisons suggest that MRI-based models, particularly those utilizing multi-parametric sequences, may outperform CT-based approaches, although direct head-to-head comparisons are limited.

### 4.3 Functional detection

Beyond direct prediction via imaging features, dynamic changes in TLS following therapeutic intervention can serve as indirect functional detection. Shu and colleagues analyzed surgical specimens after neoadjuvant immunotherapy and found that patients with high iTLS density exhibited more significant tumor regression, indicating that HCC patients with high TLS density respond better to ICIs (20). Additionally, HAIC combined with FOLFOX significantly promotes TLS formation, suggesting that post-treatment imaging re-evaluation may indirectly reflect TLS induction (44).

### 4.4 Emerging approaches

A study in renal cancer suggested that specific metabolites, such as glutathione, may be enriched in TLS (46), providing a novel direction for non-invasive HCC detection using metabolic imaging (e.g., magnetic resonance spectroscopy) or liquid biopsy. However, this approach remains experimental and requires further validation.

Despite progress, this field faces significant challenges. TLS spatiotemporal heterogeneity complicates detection; the distinct prognostic significance of iTLS and pTLS necessitates different clinical interpretations, yet existing models show weaker performance for pTLS. Most importantly, evaluation criteria for TLS maturity remain unstandardized across studies, and a validated assessment system for cross-platform, multicenter application is lacking. These issues represent core bottlenecks to clinical translation. Future work should focus on establishing unified, open image-pathology paired databases and promoting validation of deep learning models in prospective clinical trials.

## 5 Technical challenges and future directions

Although TLS demonstrate substantial clinical potential, translation from basic research to clinical application faces several key technical challenges, which also define future research priorities.

### 5.1 Standardization of spatial positioning and maturity classification

The prognostic and predictive value of TLS exhibits significant location dependence and functional heterogeneity. iTLS are generally associated with good survival outcomes and immunotherapy responses (20, 22, 58), whereas pTLS may be dysfunctional or even associated with poor prognosis (51, 52, 59). This difference stems from the variations in the composition of immune cells within it, the spatial arrangement of cells, and their maturity (23, 53). A major technical challenge is the lack of standardized spatial positioning and precise maturity classification methods. Most studies rely on manual histopathological assessment (47, 48, 52, 58), a method that is subjective, low-throughput, and cannot be applied preoperatively. Future efforts should prioritize development of automated, multi-parameter immunohistochemical/multiplex fluorescence quantitative analysis platforms integrated with artificial intelligence to achieve high-precision, reproducible TLS subtype identification and spatial quantification, thereby establishing internationally recognized TLS pathological reporting standards. Specific recommendations include: (i) adoption of consensus definitions for intratumoral versus peritumoral TLS based on objective distance criteria; (ii) standardization of maturity assessment using defined markers (presence of germinal centers, BCL6 expression, CD21^+^ FDC networks, and HEV integrity); and (iii) development of scoring systems that integrate spatial location and maturity into a unified reporting framework.

### 5.2 Non-invasive testing maturation

As iTLS status is a key predictor of prognosis and treatment response, non-invasive preoperative assessment represents a major clinical need and challenge (23, 60). Conventional CT/CECT has insufficient sensitivity and specificity to effectively distinguish TLS-related microenvironmental features (61). Multi-parameter MRI, including DCE-MRI and IVIM-DWI, has shown initial promise, with studies constructing models using tumor texture, morphology, perfusion, and diffusion features to predict iTLS presence and maturity (22, 23, 60–62). However, model performance depends on large-sample multicenter validation, and current external validation cohorts remain limited in size, resulting in weak detection capabilities for peritumoral TLS. The interpretability and biological correlation of model features also require further investigation (51). Future directions include: enhancing tissue resolution and specificity through higher-field MRI, novel contrast agents targeting TLS-associated molecules (e.g., CXCL13 or PNAd), and functional imaging sequences such as chemical exchange saturation transfer (CEST). Integrating radiomics and deep learning to explore deeper image-pathology associations; integrating radiomics with deep learning to explore deeper image-pathology associations; and developing Gd-EOB-DTPA-enhanced MRI models specifically optimized for HCC microenvironment characterization (60).

### 5.3 Addressing detection limitations through spatial multi-omics

TLS is highly dynamic and heterogeneous structures, exhibiting significant spatial heterogeneity in formation mechanisms, metabolic microenvironment characteristics (such as glutathione accumulation), immune cell composition, and interactions with surrounding matrix and tumor cells (46, 53, 63). Traditional bulk RNA sequencing or single-point tissue biopsy cannot effectively capture such spatial complexity, potentially leading to the omission or misclassification of critical biological information, such as misidentifying functionally suppressive pTLS as beneficial iTLS. Future research should leverage spatial multi-omics technologies, including spatial transcriptomics, CODEX, and MIBI-TOF, to precisely analyze cell type distribution, intercellular communication networks, and functional states within and around TLS at spatial resolution. Such approaches will elucidate core drivers of TLS formation, precise conditions for antitumor efficacy (including “dose-response” and “structure-response” relationships), and key determinants of therapeutic responsiveness.

### 5.4 Functional validation and therapeutic targeting

Although TLS, particularly iTLS, are widely regarded as “hotbeds of antitumor immunity”, their precise mechanisms of action and feasibility as therapeutic targets remain incompletely characterized (51, 52, 55, 59). Existing studies suggest that treatments such as HAIC-FOLFOX can promote the formation of TLS (43, 64). However, whether this observation translates into stable clinical benefit requires verification through large-scale randomized controlled trials (RCTS). The current technical challenges lies in the difficulty of dynamically and real-time monitoring the functional status and therapeutic intervention effects of TLS in living models or clinical settings. Future research directions include developing TLS-specific molecular imaging probes, such as PET/MR tracers targeting chemokine receptors or mature TLS markers, to achieve non-invasive and real-time dynamic monitoring of TLS. In addition, immune organoids containing immune components or humanized mouse models can be utilized to simulate the immune microenvironment of HCC, enabling screening of agent that can induce the formation of functional TLS, such as immunomodulators or targeted metabolic drugs. Concurrently, prospective clinical trials should be promoted to verify treatment and screening strategies based on TLS imaging classification or biomarkers, and to evaluate the feasibility and efficacy of the intervention approaches targeting specific pathways that regulate TLS formation. It should be emphasized that evidence for therapeutic TLS induction currently derives primarily from retrospective analyzes and small cohort studies; prospective validation is urgently required.

## 6 Summary and prospect

In summary, TLS have emerged as critical immune structures within the HCC microenvironment, with significant implications for prognosis and immunotherapy response. The key findings of this review are threefold: (i) mature intratumoral TLS serve as favorable prognostic markers and predictors of ICI response; (ii) peritumoral TLS exhibit functional heterogeneity, with conflicting prognostic associations that likely reflect differences in maturity, immune composition, and clinical context; and (iii) non-invasive detection methods, particularly MRI-based radiomics, show promise for preoperative TLS assessment, although standardization and validation are urgently needed.

Current limitations include the lack of standardized TLS classification criteria, insufficient understanding of the mechanistic drivers of TLS maturation, and the absence of prospective validation for TLS-guided treatment strategies. Addressing these gaps will require collaborative efforts across pathology, radiology, immunology, bioinformatics, and clinical medicine.

In the short term, priorities include optimizing AI-based pathological quantitative analysis workflows, promoting large-scale prospective validation of MRI/CT radiomics models, and integrating metabolic and spatial omics data to address HCC heterogeneity and non-invasive detection challenges. In the long term, efforts should focus on developing molecular imaging techniques for real-time *in vivo* TLS monitoring, constructing models that dynamically reflect the evolving immune landscape of HCC, and designing precision immunotherapy combination strategies based on TLS characteristics, such as “TLS immune typing.” The ultimate goal is to transform TLS from a descriptive immune structure into dynamic, clinically actionable biomarkers that guide individualized treatment decisions, thereby advancing the paradigm of HCC management from “group-based” to “individualized navigation” and ultimately improving patient outcomes.

## References

[1] Siegel RL Kratzer TB Giaquinto AN Sung H Jemal A . Cancer statistics, 2025. CA Cancer J Clin. (2025) 75:10–45. doi: 10.3322/caac.21871. PMID: 39817679 PMC11745215

[2] Sung H Ferlay J Siegel RL Laversanne M Soerjomataram I Jemal A . Global cancer statistics 2020: GLOBOCAN estimates of incidence and mortality worldwide for 36 cancers in 185 countries. CA Cancer J Clin. (2021) 71:209–49. doi: 10.3322/caac.21660. PMID: 33538338

[3] Vogel A Meyer T Sapisochin G Salem R Saborowski A . Hepatocellular carcinoma. Lancet. (2022) 400:1345–62. doi: 10.1016/s0140-6736(22)01200-4. PMID: 36084663

[4] Sugawara Y Hibi T . Surgical treatment of hepatocellular carcinoma. Biosci Trends. (2021) 15:138–41. doi: 10.5582/bst.2021.01094. PMID: 33746184

[5] Li Z Zhang Y Zhang B Guo R He M Liu Z-L . Bibliometric study of immunotherapy for hepatocellular carcinoma. Front Immunol. (2023) 14:1210802. doi: 10.3389/fimmu.2023.1210802. PMID: 37600802 PMC10436521

[6] Herrero A Toubert C Bedoya JU Assenat E Guiu B Panaro F . Management of hepatocellular carcinoma recurrence after liver surgery and thermal ablations: state of the art and future perspectives. Hepatobil Surg Nutr. (2024) 13:71–88. doi: 10.21037/hbsn-22-579. PMID: 38322198 PMC10839736

[7] Scheiner B Pomej K Kirstein MM Hucke F Finkelmeier F Waidmann O . Prognosis of patients with hepatocellular carcinoma treated with immunotherapy - development and validation of the CRAFITY score. J Hepatol. (2022) 76:353–63. doi: 10.1016/j.jhep.2021.09.035. PMID: 34648895

[8] Llovet JM Kelley RK Villanueva A Singal AG Pikarsky E Roayaie S . Hepatocellular carcinoma. Nat Rev Dis Primers. (2021) 7:6. doi: 10.1038/s41572-020-00240-3. PMID: 33479224 PMC13537433

[9] Wang J Chen Y Xu Y Zhang J Yang S Zhou Y . DNASE1L3-mediated PANoptosis enhances the efficacy of combination therapy for advanced hepatocellular carcinoma. Theranostics. (2024) 14:6798–817. doi: 10.7150/thno.102995. PMID: 39479454 PMC11519790

[10] Cappuyns S Llovet JM . Combination therapies for advanced hepatocellular carcinoma: Biomarkers and unmet needs. Clin Cancer Res. (2022) 28:3405–7. doi: 10.1158/1078-0432.CCR-22-1213. PMID: 35727695 PMC9614185

[11] Liu B Shi J Su R Zheng R Xing F Zhang Y . Predicting effect of anti-PD-1/PD-L1 inhibitors therapy for hepatocellular carcinoma by detecting plasma metabolite based on UHPLC-MS. Front Immunol. (2024) 15:1370771. doi: 10.3389/fimmu.2024.1370771. PMID: 38707906 PMC11067499

[12] Wang H Qian Y-W Dong H Cong W-M . Pathologic assessment of hepatocellular carcinoma in the era of immunotherapy: A narrative review. Hepatobil Surg Nutr. (2024) 13:472–93. doi: 10.21037/hbsn-22-527. PMID: 38911201 PMC11190517

[13] Chen Y Wu Y Yan G Zhang G . Tertiary lymphoid structures in cancer: maturation and induction. Front Immunol. (2024) 15:1369626. doi: 10.3389/fimmu.2024.1369626. PMID: 38690273 PMC11058640

[14] Sato Y Silina K van den Broek M Hirahara K Yanagita M . The roles of tertiary lymphoid structures in chronic diseases. Nat Rev Nephrol. (2023) 19:525–37. doi: 10.1038/s41581-023-00706-z. PMID: 37046081 PMC10092939

[15] Kim SS Sumner WA Miyauchi S Cohen EEW Califano JA Sharabi AB . Role of B cells in responses to checkpoint blockade immunotherapy and overall survival of cancer patients. Clin Cancer Res. (2021) 27:6075–82. doi: 10.1158/1078-0432.CCR-21-0697. PMID: 34230025 PMC8976464

[16] Varghese A Hess SM Chilakapati S Conejo-Garcia JR McGray AJR Zsiros E . Tertiary lymphoid structures: Exploring opportunities to improve immunotherapy in ovarian cancer. Front Immunol. (2025) 16:1473969. doi: 10.3389/fimmu.2025.1473969. PMID: 40475770 PMC12137288

[17] Peyraud F Guegan J-P Vanhersecke L Brunet M Teyssonneau D Palmieri L-J . Tertiary lymphoid structures and cancer immunotherapy: From bench to bedside. Med. (2025) 6:100546. doi: 10.1016/j.medj.2024.10.023. PMID: 39798544

[18] Yang H Chen K Meng Y Chen Z Xu Y Zhou D . Review: Radiotherapy-mediated B cells within the TLS influence the tumor microenvironment. J Immunother Cancer. (2025) 13:e011617. doi: 10.1136/jitc-2025-011617. PMID: 40664446 PMC12265816

[19] Vanhersecke L Brunet M Guégan J-P Rey C Bougouin A Cousin S . Mature tertiary lymphoid structures predict immune checkpoint inhibitor efficacy in solid tumors independently of PD-L1 expression. Nat Cancer. (2021) 2:794–802. doi: 10.1038/s43018-021-00232-6. PMID: 35118423 PMC8809887

[20] Shu DH Ho WJ Kagohara LT Girgis A Shin SM Danilova L . Immunotherapy response induces divergent tertiary lymphoid structure morphologies in hepatocellular carcinoma. Nat Immunol. (2024) 25:2110–23. doi: 10.1038/s41590-024-01992-w. PMID: 39455893 PMC12042221

[21] Ho WJ Zhu Q Durham J Popovic A Xavier S Leatherman J . Neoadjuvant cabozantinib and nivolumab converts locally advanced HCC into resectable disease with enhanced antitumor immunity. Nat Cancer. (2021) 2:891–903. doi: 10.1038/s43018-021-00234-4. PMID: 34796337 PMC8594857

[22] Long S Li M Chen J Zhong L Dai G Pan D . Transfer learning radiomic model predicts intratumoral tertiary lymphoid structures in hepatocellular carcinoma: a multicenter study. J Immunother Cancer. (2025) 13:e011126. doi: 10.1136/jitc-2024-011126. PMID: 40037925 PMC11881188

[23] Li Y Chen Y Wu Z Shi Y Li M Cai P . Noninvasive MRI imaging feature-based prediction of intratumoral tertiary lymphoid structure maturity in hepatocellular carcinoma: A multicenter retrospective study. Eur Radio. (2025) 36:1037–49. doi: 10.1007/s00330-025-11902-9. PMID: 40767869

[24] Lanickova T Hensler M Kasikova L Vosahlikova S Angelidou A Pasulka J . Chemotherapy drives tertiary lymphoid structures that correlate with ICI-responsive TCF1+CD8+ T cells in metastatic ovarian cancer. Clin Cancer Res. (2025) 31:164–80. doi: 10.1158/1078-0432.CCR-24-1594. PMID: 39163092 PMC11701433

[25] Ukita M Hamanishi J Yoshitomi H Yamanoi K Takamatsu S Ueda A . CXCL13-producing CD4+ T cells accumulate in the early phase of tertiary lymphoid structures in ovarian cancer. JCI Insight. (2022) 7:e157215. doi: 10.1172/jci.insight.157215. PMID: 35552285 PMC9309049

[26] Goronzy JJ Weyand CM . Perivascular tertiary lymphoid structures in autoimmune disease. Immunol Rev. (2025) 332:e70047. doi: 10.1111/imr.70047. PMID: 40553008 PMC12356283

[27] An D Chen G Cheng W-Y Mohrs K Adler C Gupta NT . LTβR agonism promotes antitumor immune responses via modulation of the tumor microenvironment. Cancer Res. (2024) 84:3984–4001. doi: 10.1158/0008-5472.CAN-23-2716. PMID: 39137402

[28] Cui X Gu X Li D Wu P Sun N Zhang C . Tertiary lymphoid structures as a biomarker in immunotherapy and beyond: Advancing towards clinical application. Cancer Lett. (2025) 613:217491. doi: 10.1016/j.canlet.2025.217491. PMID: 39862919

[29] Di Modugno F Di Carlo A Spada S Palermo B D’Ambrosio L D’Andrea D . Tumoral and stromal hMENA isoforms impact tertiary lymphoid structure localization in lung cancer and predict immune checkpoint blockade response in patients with cancer. EBioMedicine. (2024) 101:105003. doi: 10.1016/j.ebiom.2024.105003. PMID: 38340557 PMC10869748

[30] Zhang Y Liu G Zeng Q Wu W Lei K Zhang C . CCL19-producing fibroblasts promote tertiary lymphoid structure formation enhancing anti-tumor IgG response in colorectal cancer liver metastasis. Cancer Cell. (2024) 42:1370–1385.e9. doi: 10.1016/j.ccell.2024.07.006. PMID: 39137726

[31] Omotesho QA Escamilla A Pérez-Ruiz E Frecha CA Rueda-Domínguez A Barragán I . Epigenetic targets to enhance antitumor immune response through the induction of tertiary lymphoid structures. Front Immunol. (2024) 15:1348156. doi: 10.3389/fimmu.2024.1348156. PMID: 38333212 PMC10851080

[32] Liu W You W Lan Z Ren Y Gao S Li S . An immune cell map of human lung adenocarcinoma development reveals an anti-tumoral role of the tfh-dependent tertiary lymphoid structure. Cell Rep Med. (2024) 5:101448. doi: 10.1016/j.xcrm.2024.101448. PMID: 38458196 PMC10983046

[33] Le Rochais M Hémon P Ben-Guigui D Garaud S Le Dantec C Pers J-O . Deciphering the maturation of tertiary lymphoid structures in cancer and inflammatory diseases of the digestive tract using imaging mass cytometry. Front Immunol. (2023) 14:1147480. doi: 10.3389/fimmu.2023.1147480. PMID: 37143660 PMC10151544

[34] Bao X Lin X Xie M Yao J Song J Ma X . Mature tertiary lymphoid structures: Important contributors to anti-tumor immune efficacy. Front Immunol. (2024) 15:1413067. doi: 10.3389/fimmu.2024.1413067. PMID: 39026670 PMC11254644

[35] Yu W-W Barrett JNP Tong J Lin M-J Marohn M Devlin JC . Skin immune-mesenchymal interplay within tertiary lymphoid structures promotes autoimmune pathogenesis in hidradenitis suppurativa. Immunity. (2024) 57:2827–2842.e5. doi: 10.1016/j.immuni.2024.11.010. PMID: 39662091 PMC12404358

[36] Ware MB Wolfarth AA Goon JB Ezeanya UI Dhar S Ferrando-Martinez S . The role of interleukin-7 in the formation of tertiary lymphoid structures and their prognostic value in gastrointestinal cancers. J Immunother Precis Oncol. (2022) 5:105–17. doi: 10.36401/JIPO-22-10. PMID: 36483588 PMC9714415

[37] Li H Ding J-Y Zhang M-J Yu H-J Sun Z-J . Tertiary lymphoid structures and cytokines interconnections: The implication in cancer immunotherapy. Cancer Lett. (2023) 568:216293. doi: 10.1016/j.canlet.2023.216293. PMID: 37392991

[38] Blanchard L Girard J-P . High endothelial venules (HEVs) in immunity, inflammation and cancer. Angiogenesis. (2021) 24:719–53. doi: 10.1007/s10456-021-09792-8. PMID: 33956259 PMC8487881

[39] Tang Z Bai Y Fang Q Yuan Y Zeng Q Chen S . Spatial transcriptomics reveals tryptophan metabolism restricting maturation of intratumoral tertiary lymphoid structures. Cancer Cell. (2025) 43:1025–1044.e14. doi: 10.1016/j.ccell.2025.03.011. PMID: 40185093

[40] Qin J Gong Q Zhou C Xu J Cheng Y Xu W . Differential expression pattern of CC chemokine receptor 7 guides precision treatment of hepatocellular carcinoma. Signal Transd Targ Ther. (2025) 10:229. doi: 10.1038/s41392-025-02308-6. PMID: 40685403 PMC12277428

[41] Lu Y Yang A Quan C Pan Y Zhang H Li Y . A single-cell atlas of the multicellular ecosystem of primary and metastatic hepatocellular carcinoma. Nat Commun. (2022) 13:4594. doi: 10.1038/s41467-022-32283-3. PMID: 35933472 PMC9357016

[42] Zhao L Jin S Wang S Zhang Z Wang X Chen Z . Tertiary lymphoid structures in diseases: immune mechanisms and therapeutic advances. Signal Transd Targ Ther. (2024) 9:225. doi: 10.1038/s41392-024-01947-5. PMID: 39198425 PMC11358547

[43] Liu F Li X Zhang Y Ge S Shi Z Liu Q . Targeting tumor-associated macrophages to overcome immune checkpoint inhibitor resistance in hepatocellular carcinoma. J Exp Clin Cancer Res. (2025) 44:227. doi: 10.1186/s13046-025-03490-9. PMID: 40764998 PMC12323087

[44] Xing R Mei J Zuo Z Zou H Yu X Xu J . Enhanced formation of tertiary lymphoid structures shapes the anti-tumor microenvironment in hepatocellular carcinoma after FOLFOX-HAIC therapy. Cell Rep Med. (2025) 6(9):102298. doi: 10.1016/j.xcrm.2025.102298. PMID: 40818460 PMC12490226

[45] Qu S Jia W Liu X Yao Q Chen C Zhao Z . Intratumoral bacterial load and tertiary lymphoid structure density in hepatocellular carcinoma: association and prognostic significance. Front Immunol. (2025) 16:1652433. doi: 10.3389/fimmu.2025.1652433. PMID: 40963610 PMC12436420

[46] Arai H Sugiura Y Yamamoto S Yoshikawa T Matsuoka Y Maeda R . Glutathione synthesis via the cystine/glutamate transporter promotes the formation of tertiary lymphoid structures in the kidney. J Am Soc Nephrol. (2025) 37(2):283–98. doi: 10.1681/ASN.0000000825. PMID: 40779326 PMC12889938

[47] Jia W Yao Q Wang Y Mao Z Zhang T Li J . Protective effect of tertiary lymphoid structures against hepatocellular carcinoma: New findings from a genetic perspective. Front Immunol. (2022) 13:1007426. doi: 10.3389/fimmu.2022.1007426. PMID: 36189217 PMC9515394

[48] Ahn B Ahn H-S Shin J Jun E Koh E-Y Ryu Y-M . Characterization of lymphocyte-rich hepatocellular carcinoma and the prognostic role of tertiary lymphoid structures. Liv Int. (2024) 44:1202–18. doi: 10.1111/liv.15865. PMID: 38363048

[49] Li J Zhang L Xing H Geng Y Lv S Luo X . The absence of intra-tumoral tertiary lymphoid structures is associated with a worse prognosis and mTOR signaling activation in hepatocellular carcinoma with liver transplantation: A multicenter retrospective study. Adv Sci (Weinh). (2024) 11:e2309348. doi: 10.1002/advs.202309348. PMID: 38498682 PMC11151010

[50] Zhang H AbdulJabbar K Moore DA Akarca A Enfield KSS Jamal-Hanjani M . Spatial positioning of immune hotspots reflects the interplay between B and T cells in lung squamous cell carcinoma. Cancer Res. (2023) 83:1410–25. doi: 10.1158/0008-5472.CAN-22-2589. PMID: 36853169 PMC10152235

[51] Long S Li M Chen J Zhong L Abudulimu A Zhou L . Spatial patterns and MRI-based radiomic prediction of high peritumoral tertiary lymphoid structure density in hepatocellular carcinoma: a multicenter study. J Immunother Cancer. (2024) 12:e009879. doi: 10.1136/jitc-2024-009879. PMID: 39675785 PMC11647298

[52] Li H Liu H Fu H Li J Xu L Wang G . Peritumoral tertiary lymphoid structures correlate with protective immunity and improved prognosis in patients with hepatocellular carcinoma. Front Immunol. (2021) 12:648812. doi: 10.3389/fimmu.2021.648812. PMID: 34122408 PMC8187907

[53] Li J Xu H Han J Sun P Zhang X Wang H . Lymphocyte function in tertiary lymphoid structures predicts hepatocellular carcinoma outcome. Lab Invest. (2024) 104:102144. doi: 10.1016/j.labinv.2024.102144. PMID: 39343010

[54] Berthe J Poudel P Segerer FJ Jennings EC Ng F Surace M . Exploring the impact of tertiary lymphoid structures maturity in NSCLC: insights from TLS scoring. Front Immunol. (2024) 15:1422206. doi: 10.3389/fimmu.2024.1422206. PMID: 39376565 PMC11457083

[55] Hu L Li X Yang C Zhou B Du C Jiang N . Prognostic value of tertiary lymphoid structures in hepatocellular carcinoma: a meta-analysis and systematic review. Front Immunol. (2024) 15:1390938. doi: 10.3389/fimmu.2024.1390938. PMID: 38887293 PMC11180782

[56] Sun X Liu W Sun L Mo H Feng Y Wu X . Maturation and abundance of tertiary lymphoid structures are associated with the efficacy of neoadjuvant chemoimmunotherapy in resectable non-small cell lung cancer. J Immunother Cancer. (2022) 10:e005531. doi: 10.1136/jitc-2022-005531. PMID: 37011953 PMC9644367

[57] Jia W Zhang T Yao Q Li J Nie Y Lei X . Tertiary lymphatic structures in primary hepatic carcinoma: controversy cannot overshadow hope. Front Immunol. (2022) 13:870458. doi: 10.3389/fimmu.2022.870458. PMID: 35844587 PMC9278517

[58] Nie Y Fan H Li J Lei X Zhang T Wang Y . Tertiary lymphoid structures: associated multiple immune cells and analysis their formation in hepatocellular carcinoma. FASEB J. (2022) 36:e22586. doi: 10.1096/fj.202200269RR. PMID: 36190431

[59] Zhao R Li J Chen B Zhao J Hu L Huang K . The enrichment of the gut microbiota Lachnoclostridium is associated with the presence of intratumoral tertiary lymphoid structures in hepatocellular carcinoma. Front Immunol. (2023) 14:1289753. doi: 10.3389/fimmu.2023.1289753. PMID: 38116013 PMC10728494

[60] Li Y Li X Xiao X Cheng J Li Q Liu C . A novel hybrid model for predicting tertiary lymphoid structures and targeted immunotherapy outcomes in hepatocellular carcinoma: a multicenter retrospective study. Eur Radiol. (2025) 35:3206–22. doi: 10.1007/s00330-024-11255-9. PMID: 39658681

[61] Li P Liang Y Zeng B Yang G Zhu C Zhao K . Preoperative prediction of intra-tumoral tertiary lymphoid structures based on CT in hepatocellular cancer. Eur J Radiol. (2022) 151:110309. doi: 10.1016/j.ejrad.2022.110309. PMID: 35468444

[62] Ma L Liao S Zhang X Zhou F Geng Z Hu J . Application of intravoxel incoherent motion in the prediction of intra-tumoral tertiary lymphoid structures in hepatocellular carcinoma. J Hepatocell Carcin. (2025) 12:383–98. doi: 10.2147/JHC.S508357. PMID: 40012763 PMC11863790

[63] Gan X Dong W You W Ding D Yang Y Sun D . Spatial multimodal analysis revealed tertiary lymphoid structures as a risk stratification indicator in combined hepatocellular-cholangiocarcinoma. Cancer Lett. (2024) 581:216513. doi: 10.1016/j.canlet.2023.216513. PMID: 38036041

[64] Ma L Liao S Yuan S Li X Zhang C Zhou F . Refining risk stratification in hepatocellular carcinoma by integrating tertiary lymphoid structures and microvascular invasion: a multicenter retrospective study. Int J Surg. (2025) 111(11):8212–25. doi: 10.1097/JS9.0000000000003045. PMID: 40679983 PMC12626554

